# Ruxolitinib associated psoas muscle tuberculosis abscess in a primary myelofibrosis woman: A case report and literature review

**DOI:** 10.1097/MD.0000000000037653

**Published:** 2024-04-05

**Authors:** Chi-Yu Chen, Tun-Chieh Chen

**Affiliations:** a Department of Internal Medicine, Kaohsiung Municipal Ta-Tung Hospital, Kaohsiung Medical University, Kaohsiung, Taiwan; b Division of Infectious Diseases, Department of Internal Medicine, Kaohsiung Medical University Hospital, Kaohsiung Medical University, Kaohsiung, Taiwan; c School of Medicine, College of Medicine, Kaohsiung Medical University, Kaohsiung, Taiwan; d Center for Tropical Medicine and Infectious Disease Research and Center for Medical Education and Humanizing Health Professional Education, Kaohsiung Medical University, Kaohsiung, Taiwan.

**Keywords:** Janus kinase inhibitor, *Mycobacterium tuberculosis*, primary myelofibrosis, ruxolitinib

## Abstract

**Rationale:**

Primary myelofibrosis is a subtype of myeloproliferative neoplasm that leads to bone marrow fibrosis. Historically, the only curative option for primary myelofibrosis was allogeneic hematopoietic stem cell transplant. Ruxolitinib, a Janus kinase inhibitor, is now used for the treatment of primary myelofibrosis and polycythemia vera. It effectively improves symptoms related to splenomegaly and anemia. However, its association with the development of opportunistic infections has been observed in clinical studies and practical application.

**Patient concerns:**

A 64-year-old female with primary myelofibrosis and chronic hepatitis B infection who received ruxolitinib treatment. She was admitted for spiking fever and altered consciousness.

**Diagnosis:**

Tuberculosis meningitis was suspected but cerebrospinal fluid can’t identify any pathogens. An abdominal computed tomography scan revealed a left psoas abscess and an enlarged spleen. A computed tomography-guided pus drainage procedure was performed, showing a strong positive acid-fast stain and a positive *Mycobacterium tuberculosis* polymerase chain reaction result.

**Interventions:**

antituberculosis medications were administered. The patient developed a psoas muscle abscess caused by tuberculosis and multiple dermatomes of herpes zoster during antituberculosis treatment.

**Outcomes:**

The patient was ultimately discharged after 6 weeks of treatment without apparent neurological sequelae.

**Lessons:**

This case underscores the importance of clinicians evaluating latent infections and ensuring full vaccination prior to initiating ruxolitinib-related treatment for primary myelofibrosis.

## 1. Introduction

Primary myelofibrosis is a myeloproliferative disorder characterized by bone marrow fibrosis, splenomegaly, and anemia. Ruxolitinib, a selective inhibitor of Janus kinases (JAK1 and JAK2), received approval for the treatment of myelofibrosis in 2011 by the US Food and Drug Administration, and as a second-line therapy for patients intolerant to hydroxyurea for polycythemia vera. Notably, ruxolitinib effectively ameliorates disease-related symptoms, including splenomegaly and constitutional complaints such as fatigue, night sweats, weight loss, pruritus, and bone pain.^[1,2]^ However, the inhibition of the JAK-signal transducer and activator of transcription pathway may lead to compromised cellular immunity, resulting in immunosuppressive effects. This immunosuppression is associated with an elevated risk of opportunistic infections, including reactivation of the varicella-zoster virus (VZV), mycobacterial infections, various invasive fungal infections, and hepatitis B flares.^[3–5]^ In this report, we present a rare case of myelofibrosis complicated with a psoas muscle tuberculosis abscess and concurrent VZV reactivation, occurring after 5 months of treatment with ruxolitinib.

## 2. Case report

A 64-year-old female was admitted to our hospital in October 2020 with spiking fever and altered consciousness. She had exhibited slow responsiveness and a Glasgow Coma Scale score of E4V3M5, accompanied by incoherent speech for several days. The patient had a diagnosis of myelofibrosis and chronic hepatitis B carrier. She had been on a regimen of ruxolitinib, initially at 40 mg per day for 15 weeks, which was subsequently tapered to 20 mg per day at the time of admission.

Initial laboratory findings included: white blood cell count: 7.32 × 10^3^/μL; hemoglobin: 8.3 g/dL; myelocytes: 732 cells/μL (1%); metamyelocytes: 732 cells/μL (1%); band forms: 3660 cells/μL (5%); neutrophils: 5929 cells/μL (81%); normoblasts: 1464 cells/μL (2%). Biochemical parameters were as follows: C-reactive protein: 31.98 mg/L; procalcitonin: 1.73 ng/mL; serum glutamate pyruvate transaminase: 31.98 IU/L; ionized calcium: 4.41 mg/dL.

**Figure 1.**
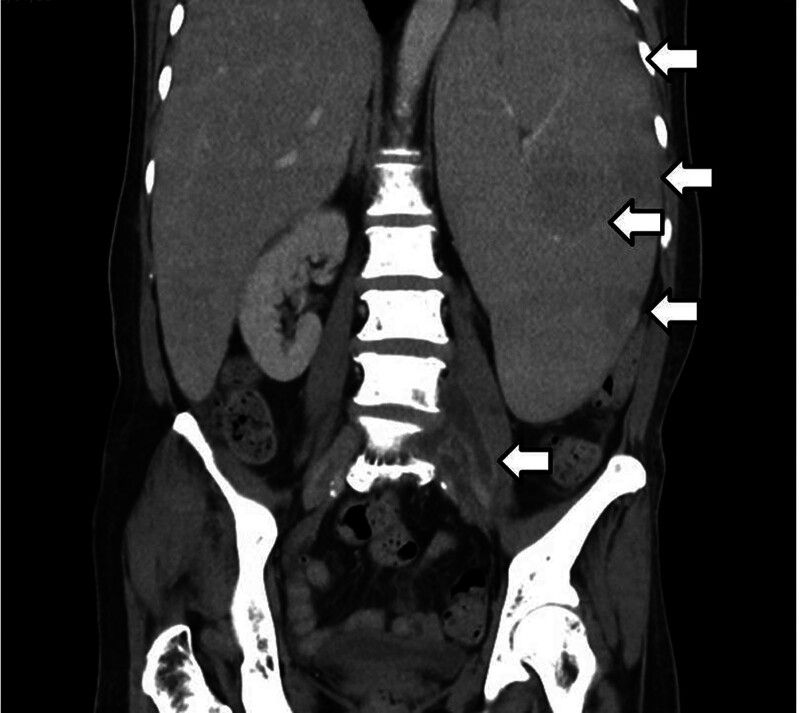
Computed tomography of left psoas abscess and splenomegaly, with multiple poor enhanced nodules. The bony structure revealed mosaic density and compatible with myelofibrosis.

Upon admission, the patient was suspected of having a central nervous system infection, and ruxolitinib treatment was temporarily suspended. Cerebrospinal fluid (CSF) analysis revealed clear with a leukocyte count of 2 cells/μL, consisting of 100% neutrophils. Glucose levels were 148 mg/dL in serum and 66 mg/dL in CSF, while protein levels were 5.4 mg/dL in serum and 22 mg/dL in CSF. Both serum and CSF tests for syphilis, *Cryptococcus neoformans*, cytomegalovirus, and toxoplasmosis returned negative results. Brain computed tomography (CT) and magnetic resonance imaging showed no specific findings. Serum lactate dehydrogenase was measured at 1264 IU/L. Viral, fungal, and bacterial cultures of CSF yielded negative results. CSF antigen tests for *Neisseria meningitidis, Streptococcus pneumoniae, Haemophilus influenzae* were all negative. A serum interferon-Gamma Release Assay (QuantiFERON) revealed a positive result. Suspecting tuberculosis meningitis, the patient was initiated on a regimen comprising rifampin 450 mg, isoniazid 250 mg, pyrazinamide 750 mg, and ethambutol 600 mg once daily. Bone marrow examination only showed hypocellular myelofibrosis without finding of mycobacterial infection as well as negative culture from sputum and CSF specimens. Dexamethasone at 15 mg per day was administered for suspected tuberculosis meningitis. Then, an abdominal CT scan revealed a left psoas abscess and an enlarged spleen (Fig. 1). A CT-guided pus drainage procedure was performed, showing a strong positive acid-fast stain and a positive *Mycobacterium tuberculosis* polymerase chain reaction result.

Ten days after commencing antituberculosis treatment, the patient developed multiple erythematous skin rashes on the right thigh. Dermatological evaluation confirmed reactivation of VZV, and treatment with acyclovir at 10 mg per kilogram body weight (42.4 kg) was prescribed every 8 hours for a 10-day course. The dosage of dexamethasone was tapered to 8 mg per day. After 21 days of antituberculosis medication, drug fever and skin rash were observed, prompting a modification of the antituberculosis regimen to include moxifloxacin 400 mg/day, and a rechallenge with isoniazid, rifampicin, and pyrazinamide. The patient was ultimately discharged after 6 weeks of treatment without apparent neurological sequelae.

## 3. Discussion

The JAK family of proteins (Jak1, Jak2, Jak3, and Tyk2) plays a pivotal role in lymphocyte proliferation, differentiation, and cytokine and interferon responses, particularly in T-cell development and proliferation. Ruxolitinib influences various immune components, including dendritic cells, natural killer cells, T helper cells, and regulatory T cells. Ruxolitinib has been shown to prolong survival and improve symptoms by inhibiting the JAK-STAT signaling pathway.^[1,6]^ However, ruxolitinib also exerts immunosuppressive effects by regulating both the innate and adaptive immune systems, leading to the suppression of proinflammatory cytokines.^[7,8]^ Consequently, this immunosuppression increases the risk of opportunistic infections, as evidenced by a recent multi-center patient-reported pilot study that included 948 unselected myeloproliferative neoplasms patients. Approximately 50.5% of these patients reported at least 1 infection episode in the span of 12 months, with upper respiratory tract infections (35.7%), herpes virus infections (15.2%), and gastrointestinal infections (14.2%) being the most common.^[9]^ Patients with myelofibrosis had a higher incidence of multiple infection episodes within a year (57.4%) compared to those with polycythemia vera (49.8%) or essential thrombocytosis (47.4%). Notably, patients receiving interferon-alpha, the JAK1/2 inhibitor (ruxolitinib), or combinations of these medications had higher rates of infection (61.4%, 68.2%, and 69.8%, respectively) compared to patients on hydroxyurea or no medications (36.9% and 55.6%, respectively). However, there was no significant difference in herpes virus infection rates between patients receiving ruxolitinib monotherapy, ruxolitinib combination therapy, or interferon therapy (25.3%, 26.4%, and 22.8%, respectively). Hospitalization rates were higher among patients receiving ruxolitinib or combined ruxolitinib therapy.^[10]^

Ruxolitinib can modulate dendritic cell function, leading to impaired CD4+ and CD8+ T-cell priming.^[7]^ Additionally, inhibition of JAK1 impairs cytokine production, resulting in an increased risk of reactivation of latent infections and potentially life-threatening opportunistic infections. A retrospective pharmacovigilance review from the US Food and Drug Administration revealed that patients receiving ruxolitinib had a higher risk of developing typical *M. tuberculosis*MTB with odds ratio 9.2 (95% CI, 7.5–11.4) and nontuberculous mycobacterial infections with odds ratio 8.3 (95% CI, 5.5–12.6). The authors concluded that patients should be carefully evaluated for latent *M. tuberculosis* and nontuberculous mycobacterial infection before starting ruxolitinib treatment. Tuberculin Skin Test or, preferably, IFN-γ Release Assay (e.g., QuantiFERON test), should be performed.^[11]^

Furthermore, it is advisable to screen patients with hematological malignancies for active or resolved hepatitis B virus (HBV) infection before initiating anticancer therapy. Patients testing positive for hepatitis B surface antigen should receive prophylactic antiviral therapy to prevent HBV reactivation during ruxolitinib-related treatment, with entecavir or tenofovir preferred over lamivudine. For patients with resolved HBV infection but a positive hepatitis B core antibody, there is no standard strategy to prevent HBV reactivation. Regular follow-up of HBV DNA levels is necessary, and antiviral therapy should be initiated upon detectable serum HBV DNA.^[12]^

Regarding VZV reactivation, primary prophylaxis is not established, but secondary prophylaxis can be considered for polycythemia vera patients receiving ruxolitinib. According to recommendations from the German Standing Committee on Vaccinations (STIKO), individuals over 60 years of age should consider vaccination against VZV with an inactivated vaccine before initiating ruxolitinib. Additionally, patients should be educated to seek immediate medical attention upon the appearance of blisters.^[13]^

## 4. Conclusion

The careful screening of latent infections and proper vaccination before initiating ruxolitinib treatment is crucial, as an increasing number of ruxolitinib-associated infections and reactivations of latent infections have been reported. Long-term studies on ruxolitinib are essential to gain a better understanding of its effects and risks in the context of prolonged treatment.

## Author contributions

**Conceptualization:** Tun-Chieh Chen.

**Visualization:** Tun-Chieh Chen, Chi-Yu Chen.

**Writing—review and editing:** Tun-Chieh Chen.

**Data curation:** Chi-Yu Chen.

**Writing—original draft:** Chi-Yu Chen.
